# Risk factors for death associated with severe influenza in children and the impact of the COVID-19 pandemic on clinical characteristics

**DOI:** 10.3389/fped.2023.1249058

**Published:** 2023-09-12

**Authors:** Qian Hu, Wen Liang, Qiuwei Yi, Yuejie Zheng, Wenjian Wang, Yuhui Wu

**Affiliations:** ^1^ Department of Respiratory Diseases, Shenzhen Children's Hospital, Shantou University School of Medicine, Shenzhen, China; ^2^ Department of Pediatric Intensive Care, Shenzhen Children's Hospital, Shantou University Medical College, Shenzhen, China

**Keywords:** influenza virus, ANE, children, ARDS, D-dimer

## Abstract

**Background:**

To summarize the clinical features of severe influenza in children and the high-risk factors for influenza-related deaths and to raise awareness among pediatricians.

**Methods:**

A retrospective study of clinical manifestations, laboratory tests, and diagnosis and treatment of 243 children with severe influenza admitted to Shenzhen Children's Hospital from January 2009 to December 2022 was conducted. Univariate logistic regression analysis and Boruta analysis were also performed to identify potentially critical clinical characteristics associated with death, and clinically significant were used in further multivariate logistic regression analysis. Subject receiver operating characteristic (ROC) curves were applied to assess the efficacy of death-related independent risk factors to predict death from severe influenza.

**Results:**

There were 169 male and 74 female patients with severe influenza, with a median age of 3 years and 2 months and 77.4% of patients under six. There were 46 cases (18.9%) in the death group. The most common pathogen was Influenza A virus (IAV) (81.5%). The most common complication in the death group was influenza-associated acute necrotizing encephalopathy (ANE [52.2%]). Severe influenza in children decreased significantly during the COVID-19 pandemic, with a median age of 5 years, a high predominance of neurological symptoms such as ANE (*P* = 0.001), and the most common pathogen being H3N2 (*P* < 0.001). D-dimer, acute respiratory distress syndrome (ARDS), and acute necrotizing encephalopathy (ANE) were significant independent risk factors for severe influenza-associated death. Furthermore, the ROC curves showed that the combined diagnosis of independent risk factors had significant early diagnostic value for severe influenza-related deaths.

**Conclusion:**

Neurological disorders such as ANE are more significant in children with severe influenza after the COVID-19 pandemic. Influenza virus infection can cause serious multisystem complications such as ARDS and ANE, and D-dimer has predictive value for early diagnosis and determination of the prognosis of children with severe influenza.

## 1. Introduction

An acute respiratory infection known as influenza is caused by the influenza virus, which has been responsible for numerous global outbreaks and pandemics (1, 2). Tens of thousands of children under 5 years of age worldwide are estimated to die yearly from influenza-related complications (2, 3). The influenza virus is highly contagious and spreads considerably fast; most infected children show mild symptoms, such as high fever, sore throat, cough, and other symptoms of upper respiratory tract infection; some progress to severe influenza due to complications such as pneumonia and toxic encephalopathy, and some severe cases progress quickly and die from acute respiratory distress syndrome (ARDS) and multiple organ failure (4).

The influenza virus is a single-stranded negative-stranded segmented RNA virus, according to the nucleoprotein, and the matrix protein is divided into four types (A, B, C, and D). Its pathogenicity and antigenicity are mainly determined by the virus envelope hemagglutinin (HA) and neuraminidase (NA), and are divided into different subtypes, such as H1N1 (5, 6). Children do not have a well-established immune system and are more susceptible to influenza virus infection, which often involves the lower respiratory tract and can cause pneumonia. Although the disease is dangerous, clinical symptoms and signs are limited in early diagnosis of severe influenza (7). Besides the respiratory system, influenza viruses can cause other systemic complications, including febrile convulsions (FS) and fulminant myocarditis. Some of these can be life-threatening or leave severe neurological sequelae, such as influenza-associated acute necrotizing encephalopathy (IANE) (8). Acute necrotizing encephalopathy (ANE) is a rare, rapidly progressive, post-infection acute severe encephalopathy named after its clinicopathological features. It has a poor prognosis and a high mortality rate, with approximately three-quarters of cases associated with children, resulting in a mortality rate of up to 30% (9, 10).

Since 2017 and especially after the COVID-19 pandemic, there has been a marked change in the epidemiological trends of influenza and other related respiratory pathogens, as well as changes in the clinical features of severe influenza (3, 11). Despite current improvements in techniques for diagnosing and treating influenza virus infections in children, the high mortality rate from severe influenza pneumonia, especially ANE, has not decreased significantly (12). This study retrospectively summarized and analyzed patients with severe influenza admitted to the Shenzhen Children's Hospital between January 2009 and December 2022 to investigate clinical characteristics and risk factors for death in children with influenza and compared the clinical characteristics of severe influenza in children after the COVID-19 pandemic. The aim is to improve pediatricians' ability to identify critical influenza cases early, take timely interventions, and improve children's prognosis.

## 2. Material and methods

### 2.1. Patients and grouping

Using retrospective analysis, 243 children with severe influenza virus infection admitted to the pediatric intensive care unit (PICU) of Shenzhen Children's Hospital from January 1, 2009, to December 31, 2022, were consecutively included and divided into 46 cases in a death group and 197 cases in a survival group according to the outcome of the children. Furthermore, using the almost initial date of the COVID-19 pandemic (January 1, 2020), as a cutoff point, the children admitted before (*n* = 198) were in the pre-pandemic group, and those admitted after (*n* = 45) were in the post-pandemic group. All children satisfied the “Expert Consensus on the Diagnosis and Treatment of Influenza in Children [2020 Edition]” (13) diagnostic criteria: epidemiological history, clinical manifestations, and positive for at least one influenza virus antigen or nucleic acid test. Clinical data collection was performed using the codes of the International Classification of Diseases, Tenth Revision (ICD 10). The study was approved by the Ethics Committee of Shenzhen Children's Hospital [ethical approval number: 2020001], and the guardians signed an informed consent form.

Inclusion criteria included: (i) confirmed severe influenza virus infection, including one or more of the following conditions: ANE, septic shock, respiratory failure, multiple organ dysfunction, and other severe clinical conditions that require monitoring and treatment; (ii) duration of ICU admission >24 h; and (iii) signed informed consent of the guardians. Exclusion criteria: (i) incomplete clinical data; (ii) combined acute pulmonary embolism and venous thrombosis at admission; and (iii) age of <1 month or >16 years.

### 2.2. Respiratory pathogens

Pharyngeal swab specimens were routinely collected from all patients on admission and immediately sent to our laboratory department for multiplex testing of respiratory pathogens (PCR capillary electrophoresis fragment analysis) (Haier Shi Gene Technology Co., Ltd, Ningbo, China), including influenza A virus (IAV), influenza B virus (IBV), H1N1, and H3N2. The operation was conducted by expert testing technicians, following the manufacturer's instructions.

### 2.3. Data collection and study variables

The data collected were verified by two specialists. The data obtained included age, sex, duration of hospitalization, history of underlying disease, clinical symptoms and signs, laboratory findings including routine blood tests, C-reactive protein (CRP), lactate dehydrogenase (LDH), alanine transferase (ALT), aspartate transferase (AST), imaging results, and treatment.

### 2.4. Statistical analysis

SPSS (v22.0, Statistical Product and Service Solutions, IBM, Chicago, IL, USA) and the Boruta package in R (v3.6.3, R Foundation for Statistical Computing, Vienna, Austria) were used to process the data. A *t*-test was used to compare continuous data that followed a normal distribution. In contrast, the Mann–Whitney *U*-test was used to compare groups of data that did not follow a normal distribution. Continuous data that did not follow a normal distribution were expressed as median *M* (interquartile range [IQR]). The chi-square test was performed to compare categorical variables between the two groups, with count data reported as the percentage of cases. There were factors that affected the regression in the study. Significant variables analyzed by univariate logistic regression at *P* < 0.1 or distinguished in the Boruta analysis were included in multivariate logistic regression to obtain independent risk factors for influenza-related deaths, plot the receiver operating characteristic (ROC) (subject work characteristic curve), and obtain the cutoff values of independent risk factors according to the Jorden index. Differences were considered statistically significant at *P* < 0.05, and all tests were two-sided.

In this study, the missing values were processed using multiple interpolation methods. The Boruta algorithm is used for variable selection before modeling. It uses a random forest model to compare existing features with inserted shadow features in successive iterations for feature selection. After some iterations, Boruta's algorithm classifies the original features into three categories “important,” “uncertain,” and “unimportant.” The result is shaded Max as the filtering index.

## 3. Results

### 3.1. Baseline and clinical characteristics

A total of 243 children with severe influenza admitted to the PICU of Shenzhen Children's Hospital from January 1, 2009, to December 31, 2022, were included in this study, with a male-to-female ratio of 2.28 (169/74) and a median age of 3 years and 2 months. Regarding age stratification, 77.4% of the children were under six, including 23.0% in the ≤1-year-old group, 23.9% in the >1–3-year-old group, and 30.5% in the >3–6-year-old group. The surviving group represented 81.7% of children under 6 years of age and the death group represented 58.7% of children under 6 years of age. The age of the death group was younger than that of the surviving group (*P* = 0.043). There were 46 cases in the death group, representing 18.9%. None of the children in the death group had been vaccinated against influenza for within 1 year before infection. Nine children, 3 in the death group and 6 in the survival group, had malnutrition, and there was no statistically significant difference in malnutrition between the two groups (*P* = 0.490). Fifty-one children were with diagnosed underlying diseases, of which 13 (28.3%) were in the death group: refractory epilepsy and complex congenital heart disease in two cases each; and immunodeficiency disorders, glycogen storage disease, nephrotic syndrome, acute lymphocyte leukemia, spinal muscular atrophy, post corneal transplantation, familial autosomal dominant necrotizing encephalopathy, Niemann-Pick disease, and hypertrophic cardiomyopathy in one case each. In the survival group, underlying diseases were reported in 38 (19.3%) cases: refractory epilepsy in six cases; nephrotic syndrome and congenital heart disease in four cases each; congenital laryngeal chondromalacia, bronchopulmonary dysplasia, glycogen storage disease, thalassemia major, bilirubin encephalopathy, delayed psychomotor development in two cases each; hypoxic ischemic encephalopathy, Pierre-Robin syndrome, congenital adrenal hyperplasia, dilated cardiomyopathy, necrotizing enterocolitis, trisomy 21 syndrome, pulmonary hypertension, phenylketonuria, leukoencephalopathy, myasthenia gravis, cerebral palsy, and Dravet syndrome in one case each. There were no statistically significant differences between the two groups in regard to underlying diseases (*P* = 0.179). The baseline characteristics of children with severe influenza are shown in Table 1.

**Table 1:** Baseline characteristics between survival and death groups.

Variables	Overall (*n* = 243)	Survival (*n* = 197)	Death (*n* = 46)	*Χ*^2^/*Z*	*P*-value
Sex				2.017	0.155
Male	169 (69.5)	141 (71.6)	28 (60.9)		
Female	74 (30.5)	56 (28.4)	18 (39.1)		
Age (months)	38.00 (14.00–70.00)	48.50 (20.00–85.30)	38.00 (13.50–63.00)	−2.020	0.043
<12	56 (23.0)	47 (23.8)	9 (19.6)		
13–36	58 (23.9)	48 (24.4)	10 (21.7)		
37–72	74 (30.5)	66 (33.5)	8 (17.4)		
>72	55 (22.6)	36 (18.3)	19 (41.3)		
Comorbidities	51 (21.0)	38 (19.3)	13 (28.3)	1.810	0.179
Malnutrition	9 (3.7)	6 (3.0)	3 (6.5)	0.477	0.490
Prematurity	23 (9.5)	18 (9.1)	5 (10.9)	0.007	0.935
Oxygen dependent	3 (1.2)	1 (0.5)	2 (4.3)	1.911	0.167

The main clinical symptoms of severe influenza were high fever (81.5%) and cough (79%), with no statistically significant differences in fever peaks in the death group compared with the survival group (*P* > 0.05). But the duration of fever was longer in the death group than in the surviving group (*P* = 0.031). Respiratory symptoms, including wet cough (42.6%) and wheezing (38.6%) were more apparent in the survivor group than in the death group (*P* < 0.05). Neurological symptoms such as coma (30.4%) were more apparent in the death group than in the survival group (*P *< 0.05). The incidence of ARDS, ANE, multiple organ dysfunction syndrome (MODS), and toxic encephalopathy was significantly higher in the death group compared with the surviving group (*P* < 0.05) (Supplementary Table S1).

### 3.2. Comparison of clinical characteristics before and during the COVID-19 pandemic

**Figure 1:**
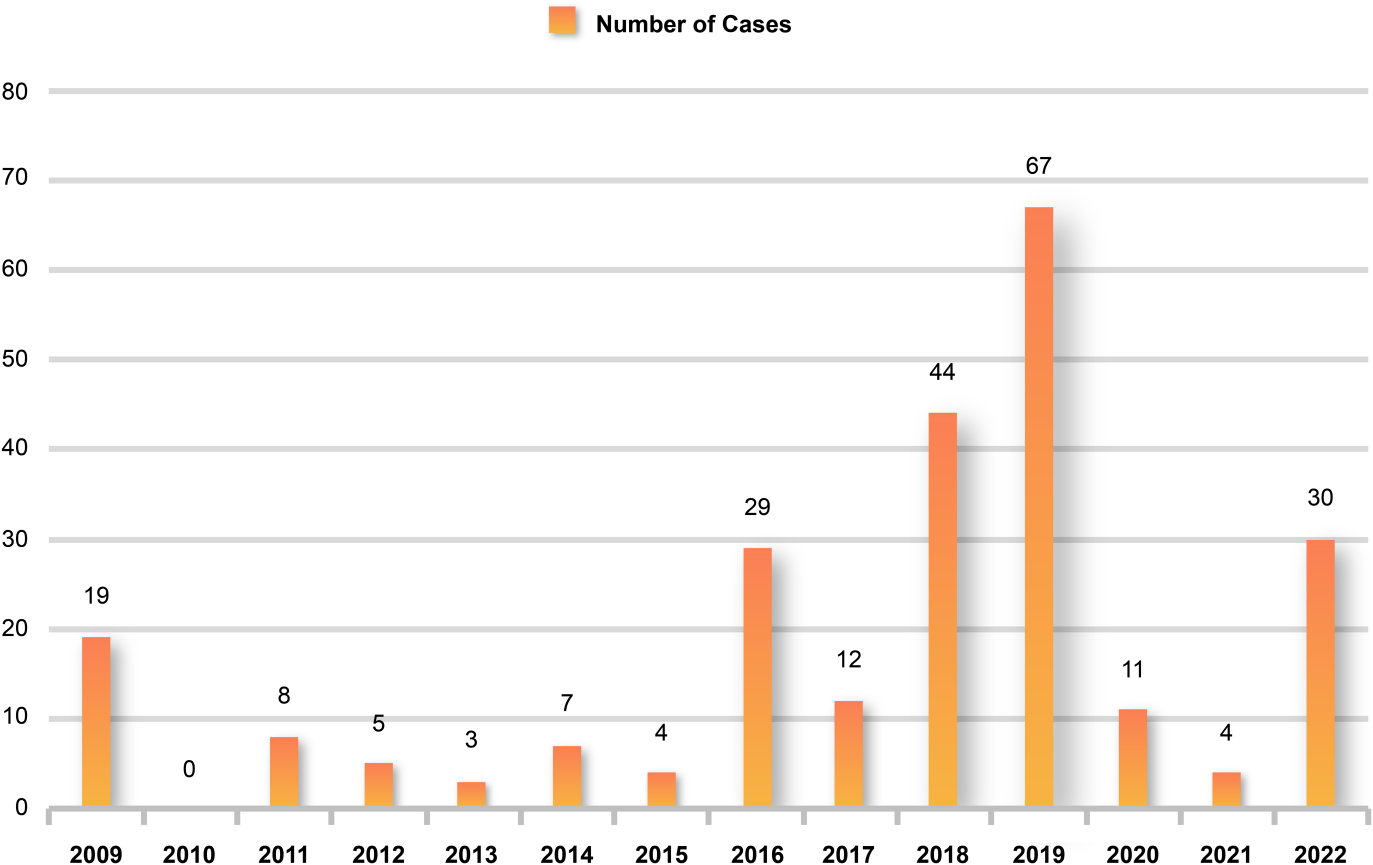
Children hospitalized for severe influenza each year. Hospital admissions for severe influenza increased each year from 2009 to 2019 but declined significantly after the start of the COVID-19 pandemic and increased again until 2022.

During the 10 years from January 2009 to December 2019, there was a trend of a yearly increase in children admitted to the PICU for severe influenza, with the highest number of 67 cases during the 2019 influenza pandemic and a significant decline in children admitted to the PICU for severe influenza during the COVID-19 pandemic, with no children admitted for severe influenza for approximately one and a half years from March 2020 to October 2021 (Figure 1). Using January 1, 2020, as the boundary, the patients were divided into a pre-group and a post-group, with 198 cases in the pre-group and 45 cases in the post-group. The median age of the patients in the post-group was 5 years old, older than the pre-group (*P* < 0.05). The clinical symptoms of children with severe influenza in the pre-group were mainly respiratory, and the incidence of cough, tachypnea, wheezing, moist rales, and pneumonia was higher than in the post-group (*P* < 0.05). In contrast, after the COVID-19 pandemic, the clinical symptoms of children with severe influenza were predominantly neurological, and the incidence of ANE was significantly higher (*P* < 0.05). Regarding pathogenicity, the H1N1 pandemic was predominant before the pandemic, whereas the H3N2 pandemic was predominant during the pandemic (Table 2). There were no significant differences in the clinical characteristics and laboratory tests of the death group before and during the COVID-19 epidemic (*P *> 0.05) (Supplementary Table S2).

**Table 2:** Comparison of clinical manifestations of severe influenza before and after the COVID-19 pandemic.

Variables	Prior (*n* = 198)	After (*n* = 45)	*Χ*^2^/*Z*	*P*-value
Sex (female)	55 (27.8)	19 (42.2)	3.612	0.057
Age (months)	37.00 (12.75–61.75)	60.00 (30.00–86.00)	−2.872	0.004
Comorbidities	31 (15.7)	20 (44.4)	18.324	<0.001
Hyperthermic	160 (80.8)	38 (84.4)	0.321	0.571
Fever duration (day)	5.00 (3.00–8.00)	4.00 (2.00–7.00)	−2.007	0.045
Cough	164 (82.8)	28 (62.2)	9.389	0.002
Tachypnea	127 (64.1)	17 (37.8)	10.556	0.001
Wheezing	80 (40.4)	6 (13.3)	11.751	0.001
Moist rales	88 (44.4)	7 (15.6)	12.852	<0.001
Coma	19 (9.6)	8 (17.8)	2.485	0.115
Seizure	46 (23.2)	16 (35.6)	2.930	0.087
ARDS	8 (4.0)	1 (2.2)	0.021	0.884
Gastrointestinal dysfunction	24 (12.1)	4 (8.9)	0.376	0.540
Toxic encephalopathy	24 (12.1)	19 (42.2)	22.811	<0.001
Myocarditis	27 (13.6)	10 (22.2)	2.094	0.148
Renal failure	8 (4.0)	7 (15.6)	6.524	0.011
ANE	43 (21.7)	21 (46.7)	11.765	0.001
Pneumonia	144 (72.7)	21 (46.7)	11.425	0.001
Plastic bronchitis	22 (11.1)	2 (4.4)	1.158	0.282
Lung consolidation	28 (14.1)	5 (11.1)	0.287	0.592
Atelectasis	32 (16.2)	6 (13.3)	0.222	0.637
White blood cell, ×10^9^/L	11.09 ± 7.08	9.82 ± 5.40	1.128	0.261
Platelet, ×10^9^/L	295.50 ± 151.17	257.82 ± 119.47	1.564	0.119
CRP, mg/L	23.27 ± 38.48	17.35 ± 19.69	1.001	0.318
ALT, U/L	19.00 (13.00–35.50)	18.50 (13.00–61.00)	−0.579	0.563
AST, U/L	44.50 (32.25–69.75)	41.00 (28.50–102.00)	−0.566	0.572
LDH, U/L	413.50 (282.25–774.00)	331.00 (262.25–586.00)	−1.795	0.073
Creatinine, μmol/L	35.75 ± 22.58	45.45 ± 45.68	−2.076	0.039
PH	7.37 (7.30–7.42)	7.39 (7.33–7.45)	−1.430	0.153
PaO_2_/FiO_2_	245.45 (179.33–371.55)	281.71 (162.46–450.00)	−1.076	0.282
APTT, sec	35.00 (30.20–41.70)	38.40 (35.40–46.00)	−2.754	0.006
PT, sec	13.20 (11.60–14.75)	14.80 (14.10–15.98)	−4.587	<0.001
D-Dimer, mg/L	0.50 (0.27–1.40)	0.74 (0.41–2.21)	−1.761	0.078
IAV	159 (80.3)	39 (86.7)	0.984	0.321
H1N1	116 (58.6)	6 (13.3)	30.035	<0.001
H3N2	8 (4.0)	26 (57.8)	87.986	<0.001
IBV	43 (21.7)	6 (13.3)	1.601	0.206
Gram-positive bacterial	28 (14.1)	7 (15.6)	0.059	0.807
Gram-negative bacterial	29 (14.6)	2 (4.4)	3.429	0.064

ARDS, acute respiratory distress syndrome; ANE, acute necrotizing encephalopathy; MODS, multiple organ dysfunction syndrome; ALT, alanine transferase; AST, aspartate transferase; CRP, C-reactive protein; LDH, lactate dehydrogenase; PH, potential of hydrogen; APTT, activated partial thromboplastin time; PT, prothrombin time; IAV, influenza A virus; IBV, influenza B virus.

### 3.3. Laboratory data, imaging, and treatment

In the death group, 35 patients were infected with the IAV (76.1%), including 30 children with H1N1 infection (65.2%), which was significantly higher than the surviving group (*P* < 0.05). There were 13 cases of influenza B virus infection in the death group (28.3%) and 2 cases of combined influenza A and B infection. Among laboratory data, AST, ALT, LDH, creatinine, and uric acid were significantly higher in the death group than the survival group (*P* < 0.05) and activated partial thromboplastin time (APTT) and Prothrombin time (PT) were significantly prolonged (*P* < 0.05). However, other laboratory data, including WBC, NC, and LC, were not statistically different between the two groups (*P* > 0.05). The rates of mixed infections of gram-positive bacteria, gram-negative bacteria, Adenovirus, Epstein–Barr virus, and Mycoplasma were not statistically different between the death and surviving groups (*P* > 0.05). There were no statistically significant differences in the incidence of chest imaging, including pleural effusion, pulmonary consolidation, and atelectasis, between the two groups (*P* > 0.05). Regarding treatment methods, there was no statistical difference between the two groups using neuraminidase inhibitors, glucocorticoids, globulin, oxygen therapy, and Extracorporeal Membrane Oxygenation (ECMO) (*P *> 0.05). Mechanical ventilation utilization was significantly higher in the death group compared to the survival group *(P *< 0.05). Antibiotics were used in 90.9% of children empirically diagnosed with bacterial infection or bacterial infection secondary to influenza before the diagnosis of influenza; however, there was no statistically significant differences between the two groups [*P *> 0.05 (Supplementary Table S3)].

### 3.4. Univariate logistic regression analysis and Boruta analysis for influenza-related mortality

**Figure 2:**
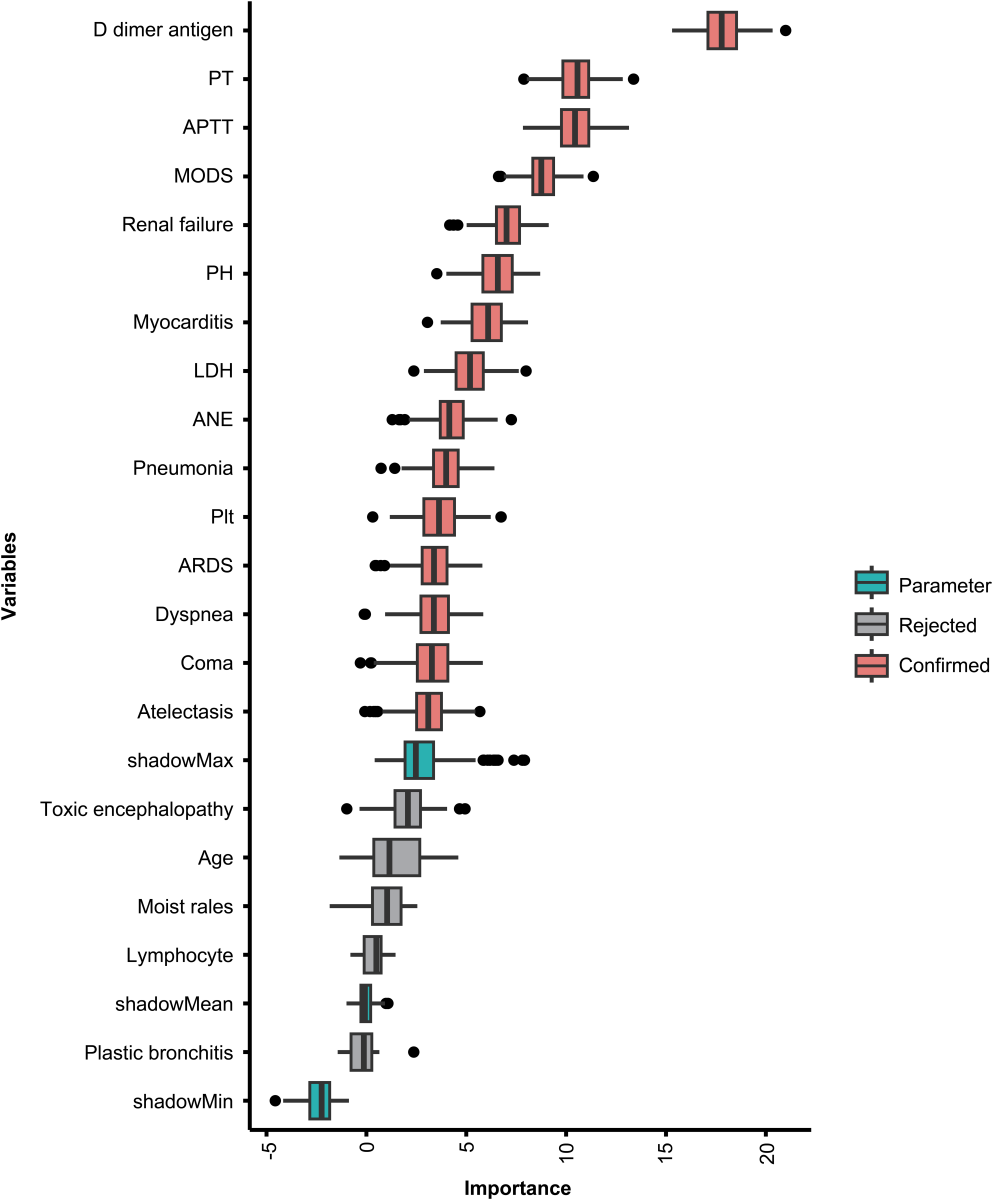
Feature importance in the Boruta feature selection process. The red boxes represent the features identified as important, the green boxes represent the Boruta parameters, and the gray boxes represent the rejected features. PT, prothrombin time; APTT, activated partial thromboplastin time; MODS, multiple organ dysfunction syndrome; PH, potential of hydrogen; LDH, lactate dehydrogenase; ANE, acute necrotizing encephalopathy; PLT, platelet; ARDS, acute respiratory distress syndrome.

Using univariate logistic regression showed age, ARDS, renal failure, ANE, pneumonia, platelet, LDH, Creatinine, potential of hydrogen (PH), D-dimer, and H1N1 infection were statistically different (*P* < 0.05), as shown in Supplementary Table S4. To further clarify clinically meaningful indicators for prediction, the Boruta algorithm was also used to screen the importance of variables. Twenty-three clinically meaningful features were evaluated. Their feature importance hierarchy is shown in Figure 2. Any other variables with importance scores below the shadow Max were eliminated; however, features with feature variable scores above the shadow Max were accepted.

### 3.5. Independent risk factors for deaths associated with severe influenza in children

**Figure 3:**
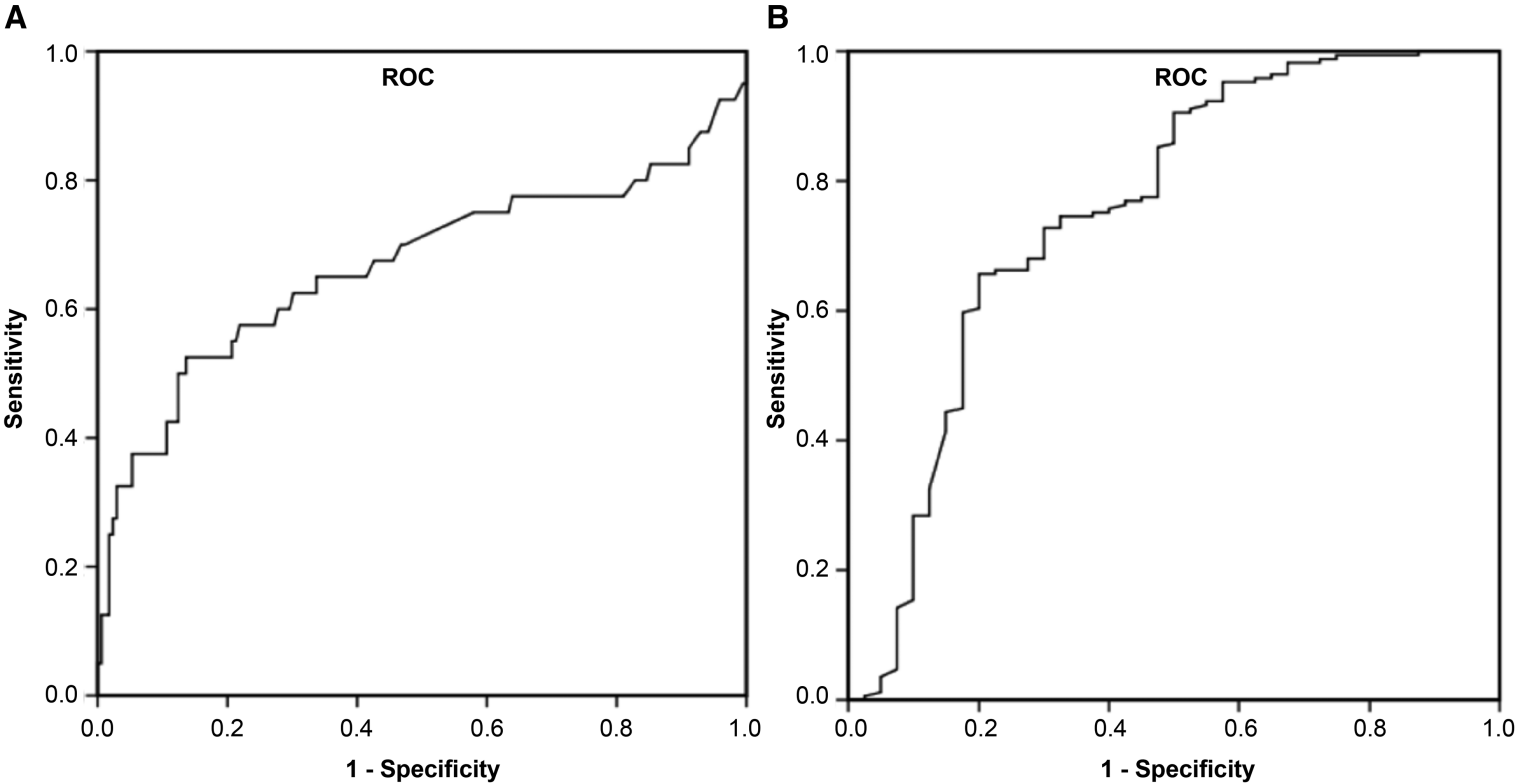
Receiver operator characteristic curve for D-dimer and combined diagnosis to predict influenza-associated death. (**A**) D-dimer, (**B**) combined diagnosis were discovered to have high AUCs by ROC analysis and could be used as biomarkers for influenza-induced death in children. AUC, area under the curve; ROC, receiver operating characteristic.

Indicators that were clinically significant in previous studies (14, 15) and had *P* < 0.1 in this study's univariate logistic regression analysis were included in the multivariate logistic regression analysis after reducing the data-based multicollinearity by using the Boruta analysis method. Three factors, ARDS, ANE, and D-dimer, were independent risk factors for severe influenza-related deaths (*P* < 0.05). The *P*-values, OR values, and 95% confidence intervals (CI) of each factor are shown in Table 3. The ROC curve was also plotted, in which D-dimer of *P* < 0.001; area under the curve (AUC) of 0.667; and 95% Cl of 0.552–0.782, indicate that D-dimer has a favorable diagnostic effect on whether death occurs in severe influenza; D-dimer at 1.95 mg/L was taken as the critical value. Its sensitivity for predicting influenza-related death was 0.525 and its specificity was 0.864. Furthermore, analysis of the combined diagnosis using ARDS, ANE, and D-dimer antigen yielded an AUC of 0.797 (*P* < 0.001), indicating that the predictive probability model also had better diagnostic accuracy with a sensitivity for predicting influenza-related deaths of 0.737 and a specificity of 0.755 (Figure 3).

**Table 3:** Multivariate logistic regression analysis of risk factors for death between survival and death groups.

Variables	Odd ratio	95% CI	*P*-value
ARDS	11.188	2.128–58.820	0.004
ANE	3.262	1.368–7.780	0.008
D-dimer	1.086	1.030–1.145	0.002

ARDS, acute respiratory distress syndrome; ANE, acute necrotizing encephalopathy.

## 4. Discussion

Influenza is a common global respiratory disease and one of the leading causes of respiratory disease death in children (16). Children's body functions are not fully developed, so the morbidity and mortality rates are higher after influenza virus infection, and children's influenza symptoms are often atypical. They can manifest themselves as a worsening of existing comorbidities, making early recognition of critical illnesses difficult, and posing a severe threat to children's health and life safety (17). This study analyzes the clinical characteristics, complementary tests, and risk factors associated with influenza-related deaths in 243 children hospitalized at the Shenzhen Children's Hospital from January 2009 to December 2022 to improve the survival quality of children with severe influenza and the early prediction ability of clinicians. We also compared the clinical characteristics of children with severe influenza before and after the COVID-19 pandemic. This study was conducted during the COVID-19 pandemic, when various respiratory pathogens, including influenza viruses, showed different epidemiological and clinical characteristics (18).

During the COVID-19 pandemic, the positive detection rate for most pathogens, such as influenza and adenovirus, decreased significantly due to various factors that transformed children's lifestyles (19, 20). This study also found a significant decrease in admissions to PICU for severe influenza during the COVID-19 pandemic, with no admissions for severe influenza for approximately 1.5 years from March 2020 to October 2021. Furthermore, changes in the clinical characteristics of children before and during the pandemic were seen, with respiratory symptoms such as cough and tachypnea being the main manifestations before the pandemic, and neurological symptoms such as ANE being the main manifestations during the COVID-19 pandemic. Moreover, ANE was an independent risk factor for severe influenza-related deaths. ANE refers to a series of clinical syndromes or acute encephalopathy syndrome accompanied by central nervous system dysfunction during acute influenza virus infection and is considered one of the significant causes of death in children with influenza, with severe consequences and poor prognosis (21). The typical symptoms are sudden onset of convulsions after acute hyperthermia, with rapid progression to coma or death within 1–2 days of onset. The disease has rapid onset and progression, with a high mortality rate in severe patients and varying degrees of neurological sequelae in survivors (22). However, the pathogenesis of ANE is still unclear, and studies have shown an association with high levels of cytokines in plasma and cerebrospinal fluid, particularly interleukin (IL)6, tumor necrosis factor (TNF)alpha, leading to a storm of cellular inflammatory factors that induce innate and inflammatory immune responses (23, 24). In Japan, ANE had the highest incidence in the 0–4 age group from 2004 to 2009, but a new change occurred during influenza A(H1N1) from 2009 to 2010, with the highest incidence in the 5–9 years age group. Furthermore, severe influenza increased after the COVID-19 pandemic compared to the pre-pandemic, with a mean age of 5 years and a predominance of H3N2 infection, consistent with previous studies (25, 26). Early awareness of ANE is difficult to identify, and targeted effective treatment measures still need to be improved. Once consciousness impairment occurs, rescue time can be lost due to the rapid progress of the disease, and research is needed for its pathogenesis and preventive measures.

In this study, D-dimer was elevated to varying degrees in children with severe influenza, with a more pronounced increase in the death group than in the survival group, the D-dimer was the first observed at 24 h after admission, rather than a peak in the disease. Children with combined acute pulmonary embolism and venous thrombosis were excluded from this study before admission. However, some patients were not reviewed for CT pulmonary angiography (CTPA) after an exacerbation, and a new thrombotic event during hospitalization could not be excluded. The D-dimer is a small molecular dimer produced by the action of fibrinolytic enzymes on cross-linked fibrin, which can be a molecular marker to determine whether the body is in a hypercoagulable or hyperfibrinolytic state (27). Various factors influence the D-dimer, and the coagulation system, including cellular (endothelial cells and platelets) and protein (coagulation factors, anticoagulants, and fibrinolytic proteases) components, are involved in the pathogenesis of influenza and COVID-19 during viral infection by enhancing viral replication and immune mechanisms (28). Endothelial cell dysfunction, severe hypoxia, and sepsis can all lead to coagulation dysfunction, and hypoxia-induced transcription factor-dependent signaling pathways lead to elevated D-dimers (28). Several studies have shown D-dimer levels to be correlated with the onset and severity of influenza pneumonia, consistent with the results of the present study (29, 30). Previous studies have shown that elevated D-dimer on admission in patients with novel coronavirus pneumonia combined with influenza virus infection is an independent predictor of death during hospitalization, noting that patients with mixed conditions of the two viruses may develop an inflammatory storm quicker than those infected with one virus (31, 32). Therefore, in viral infections, similar pathophysiological processes can lead to an elevated D-dimer associated with a poor prognosis. Close monitoring is needed to prevent deep vein thrombosis and, pulmonary embolism, and if necessary, prophylactic anticoagulation to reduce morbidity and mortality.

In healthy children, severe influenza is more common in children younger than 5 years of age, especially children younger than two. In this study, the median age of the children in the influenza-associated death group was 3 years, which was significantly lower than in the survivor group, consistent with the results of previous studies (33). However, the median age of children admitted to the PICU for severe influenza increased to 5 years after the emergence of COVID-19, which clinicians should consider. In this study, hepatic and renal functions and cardiac enzymes in the death group were significantly higher than in the survival group, suggesting different degrees of damage to multiple organ functions. Previous studies have also found that severe influenza in children can rapidly progress to ARDS, sepsis, cardiac failure, renal failure, and even MODS, with the main causes of death being respiratory complications and ANE or encephalitis (34). Therefore, coordinated multidisciplinary management of children with severe influenza is critical to controlling the early stages and avoiding uncontrolled progression and deterioration.

The clinical manifestations of children with severe influenza are atypical and changing. During the influenza season, those with respiratory symptoms or those who only show lethargy and altered consciousness should be considered as having severe influenza. Respiratory distress and convulsions are essential features of children with severe influenza. The early appearance of elevated D-dimer has a particular predictive value for the outcome of children, so clinical workers should focus on them and give treatment measures.

## 5. Conclusions

In this study, by analyzing the epidemiology and clinical characteristics of 243 children with severe influenza hospitalized in PICU for 13 years, ARDS and ANE were the main complications of childhood influenza deaths, and with the advent of COVID-19, the incidence of ANE among children with severe influenza increased significantly, and some changes in the clinical presentation of severe influenza in children occurred. Increased D-dimer have diagnostic efficacy for severe influenza-related deaths. Children with clinical manifestations should be actively tested for influenza pathogens during the pandemic season. The early combination of relevant indicators and collaborative multidisciplinary management is vital for the early control of severe influenza in children to avoid disease deterioration. However, our study has limitations. First, our study was a single-center retrospective study that included only pediatric hospitalized patients with severe influenza confirmed by pathogenic testing and did not include patients with milder symptoms treated in outpatient and emergency departments, as well as children hospitalized with general influenza. There was some bias in the study population, which affected the results. Second, our study did not examine the clinical presentation, disease severity, and prognosis of children with different subtypes of influenza virus infections and mixed COVID-19 conditions; therefore, we were unable to further elaborate on the epidemiological characteristics of influenza subtypes and mixed COVID-19 infections, and comparing different influenza subtypes and mixed infections with multiple pathogens may be more meaningful for individualized treatment. In our next steps, we will continue to establish a multicenter study to further explore the early indicators of severe influenza in children from different regions and types of virus. This will help clinicians assess children's risk and take early interventions. Finally, although this study was very rigorous in its selection of cases, as a retrospective study, several potential influences may exist. In future studies, we will design more comprehensive prospective studies to minimize confounding factors. However, the results of this preliminary study provide important clues for better understanding the clinical characteristics of very severe influenza virus infections in children, the impact of the COVID-19 epidemic on clinical manifestations, and the assessment of risk factors for death.

## Data Availability

The original contributions presented in the study are included in the article/**Supplementary Material**, further inquiries can be directed to the corresponding authors.
