# Supplementary material for: Risk factors for death associated with severe influenza in children and the impact of the COVID-19 pandemic on clinical characteristics
Source: Front Pediatr. 2023 Sep 12;11:1249058. doi: 10.3389/fped.2023.1249058 (PMC10522912; doi:10.3389/fped.2023.1249058)
Supplement: Supplementary file 1 [file Datasheet1.pdf]

Supplementary Material

Risk factors for death associated with severe influenza in children and the impact of the COVID-19 pandemic on clinical characteristics

Qian Hu[1], Wen Liang[1], Qiuwei Yi[1], Yuejie Zheng[1], Wenjian Wang[1]*, Yuhui Wu[2]*

[1] Department of Respiratory Diseases, Shenzhen Children's Hospital, Shantou University Medical College, Shenzhen, China

[2] Department of Pediatric Intensive Care Unit, Shenzhen Children's Hospital, Shantou University Medical College, Shenzhen, China

*** Correspondence:**
Wenjian Wang
wwjxx@126.com

Yuhui Wu
wyuhoo@163.com

1 Supplementary Tables

Supplementary Table S1. Clinical features between survival and death groups.

**Variables**	**Overall (n = 243)**	**Survival (n = 197)**	**Death (n = 46)**	***X^2/ Z***	***P-value***
Length of stay	9.00 (6.00–13.00)	11.00 (7.00–14.00)	4.00 (2.00–9.00)	-5.465	<0.001
Hyperthermic	198 (81.5)	162 (82.2)	36 (78.3)	0.390	0.532

Peak fever (°C)	39.50 (39.00–40.00)	39.50 (39.05–40.00)	39.20 (39.00–40.00)	-1.028	0.304
Fever duration (day)	3.00 (2.00–6.00)	3.00 (2.00–5.50)	4.00 (2.00–6.25)	-2.161	0.031
**Respiratory system**					
Cough	192 (79.0)	156 (79.2)	36 (78.3)	0.019	0.889
Wet cough	94 (38.7)	84 (42.6)	10 (21.7)	6.868	0.009
Tachypnea	144 (59.3)	122 (61.9)	22 (47.8)	3.072	0.080
Wheezing	86 (35.4)	76 (38.6)	10 (21.7)	4.625	0.032
Moist rales	95 (39.1)	82 (41.6)	13 (28.3)	2.797	0.094
Pneumonia	165 (67.9)	141 (71.6)	24 (52.2)	6.439	0.011
Plastic bronchitis	24 (9.9)	23 (11.7)	1 (2.2)	2.790	0.095
**Nervous system**					
Headache	14 (5.8)	13 (6.6)	1 (2.2)	0.653	0.419
Fatigue	28 (11.5)	18 (9.1)	10 (21.7)	5.809	0.016
Narcotism	37 (15.2)	28 (14.2)	9 (19.6)	0.828	0.363

Irritable	75 (30.9)	64 (32.5)	11 (23.9)	1.285	0.257
Seizure	62 (25.5)	51 (25.9)	11 (23.9)	0.077	0.782
Coma	27 (11.1)	13 (6.6)	14 (30.4)	21.452	<0.001
**Digestive system**					
Emesis	89 (36.6)	72 (36.5)	17 (37.0)	0.003	0.959
Diarrhea	21 (8.6)	16 (8.1)	5 (10.9)	0.094	0.760
Abdominal pain	17 (7.0)	13 (6.6)	4 (8.7)	0.033	0.856
Gastrointestinal Dysfunction	28 (11.5)	17 (8.6)	11 (23.9)	8.544	0.003
**Systemic disease**					
ARDS	9 (3.7)	4 (2.0)	5 (10.9)	5.879	0.015
Septicemia	37 (15.2)	33 (16.8)	4 (8.7)	1.875	0.171
ANE	64 (26.3)	40 (20.3)	24 (52.2)	19.523	<0.001
Congestive heart failure	17 (7.0)	6 (3.0)	11 (23.9)	21.854	<0.001
Renal failure	15 (6.2)	5 (2.5)	10 (21.7)	20.539	<0.001

Air leak syndrome	21 (8.6)	19 (9.6)	2 (4.3)	0.739	0.390
MODS	18 (7.4)	5 (2.5)	13 (28.3)	32.323	<0.001
Toxic encephalopathy	43 (17.7)	27 (13.7)	16 (34.8)	11.375	0.001

Abbreviations: ARDS, acute respiratory distress syndrome; ANE, acute necrotizing encephalopathy; MODS, multiple organ dysfunction syndrome.

Supplementary Table S2. Comparison of clinical manifestations of death group before and during the COVID-19 pandemic.

**Variables**	**Prior**	**After**	***X^2/ Z***	***P-value***
Sex (female)	15 (41.7)	3 (30.0)	0.092	0.762
Age (months)	38.00 (12.50–82.75)	74.50 (36.75–119.75)	-1.918	0.056
Comorbidities	10 (27.8)	3 (30.0)	0.000	1.000
Hyperthermic	28 (77.8)	8 (80.0)	0.000	1.000
Fever duration (day)	4.00 (2.00–6.75)	3.00 (2.00–5.25)	-0.904	0.366
Cough	29 (80.6)	7 (70.0)	0.080	0.777
Tachypnea	19 (52.8)	3 (30.0)	0.842	0.359
Wheezing	9 (25.0)	1(10.0)	0.341	0.559

Moist rales	12 (33.3)	1 (10.0)	1.108	0.292
Coma	10 (27.8)	4(40.0)	0.126	0.723
ARDS	4 (11.1)	1 (10.0)	0.000	1.000
MODS	9 (25.0)	4 (40.0)	0.286	0.593
Toxic encephalopathy	12(33.3)	4 (40.0)	0.000	0.987
Myocarditis	10 (27.8)	1 (10.0)	0.558	0.455
Renal failure	6 (16.7)	4(40.0)	1.321	0.250
ANE	18 (50.0)	6(60.0)	0.041	0.840
Pneumonia	20 (55.6)	4(40.0)	0.264	0.608
Lung consolidation	2 (5.6)	1 (10.0)	0.00	1.000
White blood cell, $\times 10^{9}$/L	8.59 (7.80–12.34)	9.97 (4.69–22.38)	-0.133	0.894
Platelet, $\times 10^{9}$/L	197.50 (137.50–294.75)	195.50 (143.25–304.25)	-0.107	0.915
CRP, mg/L	8.15 (1.68–27.68)	12.07 (5.79–26.74)	-0.812	0.417
ALT, U/L	34.00 (15.50–210.50)	136.00 (21.50–475.25)	-0.650	0.516

AST, U/L	67.00 (38.00–584.00)	220.00 (23.50–524.25)	-0.082	0.935
LDH, U/L	710.00 (325.00–1194.00)	503.5 (371.75–997.00)	-0.710	0.478
PH	7.29 (7.20–7.40)	7.34 (7.26–7.43)	-0.519	0.604
APTT, sec	44.00 (33.40–55.80)	39.15 (34.50–47.25)	-0.499	0.618
PT, sec	14.50 (12.40–18.00)	16.00 (14.40–17.80)	-0.858	0.391
D-dimer , mg/L	0.91 (0.23–11.10)	2.48 (1.52–37.66)	-1.792	0.073
IAV	29 (80.6)	6(40.0)	0.863	0.353
H1N1	28 (77.8)	2 (20.0)	9.111	0.003
IBV	9 (25.0)	4(40.0)	0.286	0.593

Abbreviations: ARDS, acute respiratory distress syndrome; ANE, acute necrotizing encephalopathy; MODS, multiple organ dysfunction syndrome; ANE, acute necrotizing encephalopathy; IAV, Influenza A virus; IBV, Influenza B virus; PH, potential of hydrogen; APTT, activated partial thromboplastin time; PT, prothrombin time.

Supplementary Table S3. Laboratory findings and imaging characteristics between survival and death groups.

Variables	Overall (n = 243)	Survival (n = 197)	Death (n = 46)	X^2/ Z/t	*P-value*
White blood cell, $\times 10^9$/L	10.85 ± 6.81	10.81 ± 6.56	11.04 ± 7.86	0.209	0.835
Neutrophils, $\times 10^9$/L	7.78 ± 6.13	7.80 ± 6.13	7.67 ± 6.23	-0.133	0.894
Lymphocyte, $\times 10^9$/L	1.51 (0.80–3.12)	1.46 (0.82–2.92)	1.61 (0.71–3.98)	-0.508	0.611
Hemoglobin, g/L	111.37 ± 27.88	112.51 ± 28.96	106.50 ± 22.29	-1.318	0.189
Platelet, $\times 10^9$/L	288.52 ± 146.34	304.08 ± 145.74	221.89 ± 130.57	-3.509	0.001
CRP, mg/L	22.15 ± 35.74	22.64 ± 36.09	19.97 ± 34.48	-0.447	0.655
PCT, mg/L	0.48 (0.10–3.12)	2.50 (0.28–29.56)	0.38 (0.09–1.86)	-2.918	0.004
ALT, U/L	19.00 (13.00–39.00)	18.00 (13.00–30.00)	42.00 (16.50–220.00)	-3.591	<0.001
AST, U/L	43.00 (31.50–70.50)	42.00 (31.00–64.00)	124.00 (37.50–565.00)	-3.543	<0.001
LDH, U/L	388.50 (277.00–739.50)	357.00 (273.00–655.00)	604.00 (332.50–1,049.83)	-3.328	0.001

CK, U/L	184.00 (92.50–533.50)	168.00 (90.30–507.00)	300.00 (96.30–723.00)	-1.536	0.124
CK-MB, μg/L	21.04 ± 103.60	18.01 ± 92.68	33.89 ± 141.48	0.925	0.356
Creatinine, μmol/L	37.56 ± 28.48	34.83 ± 24.51	49.44 ± 39.79	3.160	0.002
Uric acid, mmol/L	3.60 (2.56–4.99)	3.32 (2.51–4.68)	4.70 (2.85–7.74)	-2.614	0.009
PH	7.38 (7.30–7.42)	7.38 (7.32–7.43)	7.34 (7.20–7.41)	-2.896	0.004
PaO_2/FiO_2	250.00 (176.06–381.82)	253.03 (101.82–391.73)	242.00 (124.39–380.56)	-1.119	0.263
APTT, sec	36.25 (30.90–42.00)	35.60 (30.30–40.60)	42.00 (33.40–55.20)	-3.998	<0.001
PT, sec	13.60 (12.00–15.20)	13.30 (11.95–14.75)	15.45 (12.58–17.85)	-3.701	<0.001
D-Dimer, mg/L	0.53 (0.31–1.48)	0.50 (0.30–1.01)	2.12 (0.42–14.08)	-3.288	0.001
Mixed infection					
Gram-positive bacterial	35 (14.4)	31 (15.7)	4 (8.7)	1.499	0.221
Gram-negative bacterial	31 (12.8)	26 (13.2)	5 (10.9)	0.182	0.670

Adenovirus	6 (2.5)	4 (2.0)	2 (4.3)	0.148	0.701
Epstein–Barr virus	11 (4.5)	9 (4.6)	2 (4.3)	0.004	0.948
Mycoplasma	24 (9.9)	21 (10.7)	3 (6.5)	0.328	0.567
IAV	198(81.5)	163 (82.9)	35 (76.1)	1.094	0.296
H1N1	122 (50.2)	92 (46.7)	30 (65.2)	5.115	0.024
H3N2	34 (14.0)	30 (15.2)	4 (8.7)	1.348	0.246
IBV	49 (20.2)	36 (18.4)	13 (28.3)	2.358	0.133
Pleural effusion	47 (19.3)	36 (18.3)	11 (23.9)	0.760	0.383
Lung consolidation	33 (13.6)	30 (15.2)	3 (6.5)	2.409	0.121
Atelectasis	38 (15.6)	27 (13.7)	11 (23.9)	2.945	0.086
Treatment					
Neuraminidase inhibitor	237 (97.5)	192 (97.5)	45(97.8)	0.000	1.000

Oxygen therapy	233(95.9)	187 (94.9)	46 (100.0)	1.319	0.251
Mechanical ventilation	107 (44.0)	61 (31.0)	46 (100.0)	69.345	<0.001
ECMO	4 (1.6)	2 (1.0)	2 (4.3)	0.914	0.339
Glucocorticoid	146 (60.1)	118 (59.9)	28 (60.9)	0.015	0.904
Globulin	129 (53.1)	105 (53.3)	24 (52.2)	0.019	0.890
Antibiotic	221 (90.9)	179 (90.9)	42 (91.3)	0.009	0.925

Abbreviations: ARDS, acute respiratory distress syndrome; ANE, acute necrotizing encephalopathy; MODS, multiple organ dysfunction syndrome; ALT, alanine transferase; AST, aspartate transferase; CRP, C-reactive protein; LDH, lactate dehydrogenase; PH, potential of hydrogen; APTT, activated partial thromboplastin time; PT, prothrombin time; IAV, Influenza A virus; IBV, Influenza B virus; PCT, procalcitonin; CK, creatine Kinase; CK-MB, creatine kinase-MB; ECMO, Extracorporeal Membrane Oxygenation.

Supplementary Table S4. Univariate logistic regression analysis between survival and death groups.

**Variables**	**Odd ratio**	**95% CI**	***P*-value**
Sex	0.618	0.317–1.205	0.158
Age (months)	0.990	0.982–0.998	0.013

Comorbidities	1.648	0.792–3.430	0.181
Hyperthermic	0.935	0.711–1.228	0.533
Fever duration (day)	1.091	0.995–1.197	0.065
Cough	0.946	0.434–2.065	0.889
Tachypnea	0.564	0.295–1.075	0.082
Moist rales	0.552	0.274–1.114	0.097
Seizure	0.900	0.426–1.902	0.782
ARDS	5.884	1.514–22.864	0.010
Gastrointestinal Dysfunction	3.328	1.436–7.711	0.005
Toxic encephalopathy	3.358	1.618–6.969	0.001
Myocarditis	3.849	1.801–8.225	0.001
Renal failure	10.667	3.442–33.053	<0.001

ANE	4.282	2.181–8.407	<0.001
Pneumonia	0.433	0.225–0.835	0.012
Plastic bronchitis	0.168	0.022–1.278	0.085
Atelectasis	1.979	0.898–4.359	0.090
White blood cell, $\times 10^{9}$/L	0.995	0.950–1.042	0.834
Lymphocyte, $\times 10^{9}$/L	0.887	0.780–1.008	0.067
Platelet, $\times 10^{9}$/L	1.005	1.002–1.008	0.001
C-reactive protein, mg/L	1.002	0.992–1.012	0.654
Alanine aminotransferase, U/L	0.999	0.998–1.000	0.016
Aspartate aminotransferase, U/L	0.999	0.999–1.000	0.007
Lactate dehydrogenase, U/L	1.000	1.000–1.001	0.014
Creatinine, μmol/L	0.986	0.976–0.996	0.008

PH	163.837	7.002–3,833.823	0.002
PaO_2/FiO_2	1.001	0.999–1.003	0.356
APTT, sec	0.945	0.915–0.976	0.001
PT, sec	0.859	0.790–0.934	<0.001
D-dimer, mg/L	0.917	0.873–0.963	<0.001
IAV	1.253	0.136–11.556	0.842
H1N1	0.110	0.026–0.458	0.002
H3N2	0.325	0.064–1.641	0.174
IBV	0.128	0.015–1.107	0.062

Abbreviations: ARDS, acute respiratory distress syndrome; ANE, acute necrotizing encephalopathy; MODS, multiple organ dysfunction syndrome; CI, confidence interval; ANE, acute necrotizing encephalopathy; IAV, Influenza A virus; IBV, Influenza B virus; PH, potential of hydrogen; APTT, activated partial thromboplastin time; PT, prothrombin time.
